# Edible Bird’s Nest, an Asian Health Food Supplement, Possesses Moisturizing Effect by Regulating Expression of Filaggrin in Skin Keratinocyte

**DOI:** 10.3389/fphar.2021.685982

**Published:** 2021-07-20

**Authors:** Queenie Wing Sze Lai, Maggie Sui Sui Guo, Kevin Qiyun Wu, Zhitao Liao, Dongshi Guan, Tina Tingxia Dong, Penger Tong, Karl Wah Keung Tsim

**Affiliations:** ^1^ Shenzhen Research Institute, The Hong Kong University of Science and Technology, Shenzhen, China; ^2^ Division of Life Science and Center for Chinese Medicine R and D, The Hong Kong University of Science and Technology, Hong Kong, China; ^3^ Department of Physics, The Hong Kong University of Science and Technology, Hong Kong, China; ^4^ State Key Laboratory of Nonlinear Mechanics, Institute of Mechanics, Chinese Academy of Sciences, Beijing, China

**Keywords:** edible bird’s nest, sialic acid, moisturizing, filaggrin, filaggrin-2, atomic force microscopy

## Abstract

Edible bird’s nest (EBN) has been consumed as a Chinese delicacy for hundreds of years; the functions of which have been proposed to prevent lung disease, strengthen immune response, and restore skin youthfulness. To support the skin function of EBN, the water extract and the enzymatic digest of EBN with enriched digested peptides were tested in cultured keratinocyte, HaCaT cell line. The effects of EBN extract and digest in inducing proteins crucial for skin moisturizing were determined in both *in vitro* and *ex vivo* models. In cultured keratinocytes, the expressions of S100-fused type proteins contributing to skin barrier function in the stratum corneum, e.g. filaggrin and filaggrin-2, were determined in both mRNA and protein levels, which were markedly induced in the treatment of EBN extract or digest. The EBN-induced gene transcriptions of filaggrin and filaggrin-2 were mediated by activation of p38 MAPK pathway and various transcription factors, e.g. GATA3, PPARα, PPARβ, and PPARγ: these transcriptional factors were markedly activated by the digested products of EBN, as compared to the extract, in cultured keratinocytes. By using atomic force microscopy (AFM), the EBN-treated keratinocyte was shown to have more liquid-like morphology, as compared to a control cell. The EBN digest showed better induction on these moisturizing effects as compared to the extract. These lines of evidence therefore suggested the water moisturizing effect of EBN in skin function.

## Introduction

Edible bird’s nest (EBN; Yan Wo), the solidified saliva secreted by the swiftlets, *Aerodramus fuciphagus*, has been consumed in Asia for several hundred years as a delicacy. Today, the majority of EBN is produced by domesticated swiftlets in specialized houses from Indonesia, Malaysia, Vietnam, and Thailand. According to historical literatures in Chinese medicine, EBN was recorded to prevent lung diseases, to reduce aging and to improve complexion (Wong, 2013). In line with the historical records, several lines of evidence have suggested the functions of EBN in improving immune response (Zhao et al., 2016), encouraging cell regeneration (Roh et al., 2012), promoting anti-oxidation (Ghassem et al., 2017) and facilitating skin whitening (Chan et al., 2015).

EBN contains a rich source of protein, having over 50% by dry weight, which provides a rich source of glycoproteins and amino acids for human consumption (Halimi et al., 2014; Wong et al., 2018b). Typically, the consumption of EBN is by extensive cooking; but this process may degrade the active ingredients for its nutritious values, such as those small peptides specific for skin regenerating functions. EBN exists as a complex macromolecular form that is, not easily transformed into a dietary supplement or as a skin-care product. Therefore, we have developed methods to maximize the extracting efficiency of protein/peptide in EBN, as well as the soluble sialic acids (Wong et al., 2018a). Having the developed method for EBN extraction, the extractable protein/peptide was significantly increased by 70–90%.

In the skin’s surface, the epidermal layer stratum corneum retains water by those hygroscopic agents, named as natural moisturizing factors (NMFs), which are presented in corneocytes and orderly arranged intercellular lipids forming a barrier against trans-epidermal water loss (TEWL) and maintaining water balance of skin. Over 70% of NMFs are synthesized and derived from filaggrin. Filaggrin (filament aggregating protein) is a filament-associated protein that binds to keratin fibers in epithelial cells to prevent TEWL (Sandilands et al., 2009; Vestergaard and Deleuran, 2014). 10–12 filaggrin units are post-translationally hydrolyzed from a large profilaggrin precursor protein during terminal differentiation of epidermal cells (Sandilands et al., 2007). In humans, profilaggrin is encoded by the filaggrin gene, which is part of the S100 fused-type protein family within the epidermal differentiation complex on chromosome 1q21. Besides filaggrin, filaggrin-2, a closely related member of filaggrin family, also contributes to the formation of NMFs (Hoste et al., 2011). Both filaggrin and filaggrin-2 are protein markers of late differentiation of the epidermal cells, localizing in keratohyalin granules at stratum granulosum. Under the proteolysis process, filaggrin and filaggrin-2 are dephosphorylated, deiminated, and finally proteolyzed into NMFs at the stratum corneum (Sandilands et al., 2009; Hsu et al., 2011). Major NMFs formed from filaggrin are histidine, urocanic acid, and pyrrolidone carboxylic acid (Sandilands et al., 2009). In line with this hypothesis, the loss-of-function mutation of filaggrin is the major genetic risk factor of atopic dermatitis; while the reduced expression of filaggrin-2 is closely correlated to psoriatic lesions (Kypriotou et al., 2012). Both proteins are controlling epithelial homeostasis and protecting the skin barrier (Sandilands et al., 2009).

EBN is consumed as soup, or can be applied as external skin-care products, to improve complexion *via* its moisturizing, whitening, and anti-oxidation effects. Despite the recognized moisturizing effect of EBN in skin, the mechanistic action of EBN as a moisturizer has not been illustrated. The moisturizing effect of EBN has been proposed to be originated from polar interactions between sialic acids in EBN and water molecules (Ng, 1991); however, the detailed experimental data has not been shown. Here, we are attempting to demonstrate the moisturizing effect of EBN, both in water extract and enzymatic digested products, by revealing the expressions of filaggrin and filaggrin-2 by real-time PCR analysis, western blot analysis, and immunofluorescence analysis in EBN-treated cultured keratinocytes. Besides, the EBN-induced signaling cascade in activating expression of filaggrin was illustrated here. The moisturizing effect of EBN was further demonstrated in skin *ex vivo* culture, as well as the measurement of cell moisturization of EBN-treated keratinocytes by atomic force microscopy (AFM).

## Materials and Methods

### Preparation of EBN Extract and Digestion

White EBN, originated from Malaysia house production site, was purchased in Hong Kong market: the sample was of the standard “cup” grade. The sample was stored at room temperature. The raw EBN material was first weighed and soaked in 1:100 (w/v) double deionized (DDI) water overnight for expansion. On the following day, the expanded EBN was rinsed with DDI water three times to remove water-soluble inorganics. The softened EBN was boiled at 98 ± 2°C with 1:30 (w/v) DDI water for 8 h under constant stirring. The stewed EBN was filtered, and the filtrate was collected for lyophilization. The lyophilized filtrate powder was considered as EBN extract. The dried EBN extract was weighed and digested with 1:100 (w/v) simulated gastric fluid (SGF; without enzyme; containing 0.07 M hydrochloric acid and 0.1 M sodium chloride, catalog number: 01651) and 7.6% (w/w) pepsin (from porcine gastric mucosa lyophilized powder, ≥ 2,500 units/mg protein, catalog number: P7012) (Sigma-Aldrich, St Louis, United States) for 48 h at 37°C for complete digestion. After 48 h, the digestion was terminated by neutralizing nine parts of digested solution with one part of 0.7 M sodium hydroxide solution. Finally, lyophilization was performed to prepare the EBN digest.

### HPLC Analysis

HPLC-UV chromatographic separation of EBN digest was performed on an Agilent HPLC 1200 series system (Agilent, Waldbronn, Germany) equipped with a diode array detector (DAD). The EBN digest was reconstituted in DDI water and separated by Superdex 75 gel filtration column (catalog number: GE17-5174-01) (GE Healthcare, Little Chalfont, United Kingdom). UV absorbance was measured at wavelength 214 and 280 nm for peptide and protein detection, respectively. Samples were eluted with phosphate-buffered saline (PBS) mobile phase (containing 0.14 M NaCl, 2.68 mM KCl, 0.01 M Na_2_HPO_4_, and 1.76 mM KH_2_PO_4_) at a flow of 0.4 mL/min. Protein markers consisting of α-melanocyte-stimulating hormone (1.7 kDa) (Sigma-Aldrich, catalog number: M4135-1 MG), aprotonin (6.5 kDa), ribonuclease A (13.7 kDa), carbonic anhydrase (29 kDa), ovalbumin (43 kDa), conalbumin (75 kDa), and blue dextran (> 2,000 kDa) (GE Healthcare, catalog number: 28-4038-41) were employed for size estimation.

### Animals and Cell Culture

C57BL/6 mice were supplied by Animal and Plant Care Facility in The Hong Kong University of Science and Technology (HKUST). All experiments were performed according to the guidelines of Department of Health, The Government of Hong Kong SAR. The experimental procedures had been reviewed and approved by the Animal Ethics Committee of the university (Reference No.: (20–104) in DH/HT&A/8/2/2 Pt.2). Housing was maintained at a constant temperature and humidity, under a fixed 12-h light/dark cycle and free access to food and water. Human epidermal keratinocytes, HaCaT cells (AddexBio, San Diego, United States, catalog number: T0020001), were cultured in Dulbecco’s modified Eagle’s medium (DMEM) (catalog number: 1280017), or custom made DMEM without calcium chloride, phenol red, nor sodium bicarbonate (catalog number: 31600034), supplemented with 10% (v/v) fetal bovine serum (FBS) (catalog number: 10270-106) and 1% (v/v) penicillin/streptomycin (10,000 U and 10,000 μg/mL) (catalog number: 15140-122) in a humidified atmosphere with 5% CO_2_ at 37°C. All culture reagents were purchased from Thermo Fisher Scientific (Waltham, United States).

### DNA Transfection

The DNA construct pFLG2-eGFP was composed of the vector pEGFP-N1 (Addgene, Watertown, United States, catalog number: 6085-1) containing a filaggrin-2 promoter, which drives the transcription of a red-shifted variant of wild-type green fluorescence protein reporter. Cultured HaCaT keratinocytes were transfected using jetPRIME reagent (Polyplus Transfection, New York, United States). In short, a transfection mix containing jetPRIME buffer (catalog number: 712-60), DNA construct and jetPRIME reagent (catalog number: 114-15) were added to cell culture and incubated overnight, followed by transferring transfected cells to a 96-well clear-bottom black plate. Seeded at 1 × 10^5^ cells/mL. Cells were treated with or without EBN samples, including extract and digest. CaCl_2_ (Sigma-Aldrich, catalog number: 31307-500G) at 0.16 mM was used as a positive control. Samples were examined by Nikon fluorescence microscope. Green fluorescent protein (GFP) quantification was performed in a FlexStation^®^ 3 Benchtop Multi-Mode Microplate Reader (Molecular Devices, Sunnyvale, United States). Fluorescence signals were measured under excitation/emission wavelength 488/509 nm. GFP activities were normalized to total proteins using Bradford assay (Bio-Rad Laboratories, Hercules, United States, catalog number: 5000006), and the values were expressed as percent increase of transfected untreated control.

### Real-Time PCR Analysis

Through real-time PCR analysis, the amounts of filaggrin and filaggrin-2, GATA3, PPARα, PPARβ, PPARγ, CASP14, and GAPDH mRNAs were quantified. HaCaT keratinocytes were grown in a 12-well culture plate in a concentration of 1.5 × 10^5^ cells/mL. EBN samples, including extract and digest, were applied. CaCl_2_ (Sigma-Aldrich, catalog number: 31307-500G) at 0.16 mM (positive control of filaggrin and filaggrin-2), IL-4 (Thermo Fisher Scientific, catalog number: A42601) at 100 ng/mL (positive control of GATA3), linoleic acid (Sigma-Aldrich, catalog number: 62230-5ML-F) at 30 mM (positive control of PPARs), and 1α, 25-dihydroxycholecalciferol (vitamin D_3_) (Sigma-Aldrich, catalog number: C9758-5G) at 10^–5^ M (positive control of CASP14) were applied. Total RNA was extracted after 24 h of EBN application, using RNAzol^®^ RT RNA isolation reagent (Molecular Research Center, Cincinnati, United States, catalog number: RN190). The RNA quality and amount were determined by NanoDrop™ (Thermo Fisher Scientific) measurements at A260/A280 and A260/A230. The extracted total RNA sample was normalized to 2 μg and any contaminating genomic DNA was removed with DNase I (New England Biolabs, Hitchin, United Kingdom, catalog number: M0303S), according to the manufacturer’s protocol. 500 ng RNA sample was reverse transcribed to cDNA using PrimeScript™ RT Reagent Kit (TaKaRa, Kusatsu, Japan, catalog number: RR036A), according to the manufacturer’s protocol. Relative gene quantification was performed by LightCycler^®^ 480 Real-Time PCR System (Roche, Basel, Switzerland). The sequences of specific primers for filaggrin and filaggrin-2, GATA3, PPARα, PPARβ, PPARγ, CASP14, and GAPDH were shown as followed: sense 5′-GCT GAA GGA ACT TCT GGA AAA GG-3′ and antisense 5′- GTT GTG GTC TAT ATC CAA GTG ATC-3′ for filaggrin; sense 5′-CTG TGG TCA TTC ATG GAG TGG-3′ and antisense 5′-CCC TAG AAG GGC TAA TGT GTG A-3′ for filaggrin-2; sense 5′-GCG GGC TCT ATC ACA AAA TGA-3′ and antisense 5′-GCC TTC GCT TGG GCT TAA T-3′ for GATA3; sense 5′-GCA CTG GAA CTG GAT GAC AG-3′ and antisense 5′-TTT AGA AGG CCA GGA CGA TCT-3′ for PPARα; sense 5′-CAG AAG AAG AAC CGC AAC A-3′ and antisense 5′-CGC CAT ACT TGA GAA GGG T-3′ for PPARβ; sense 5′-CAG GAA AGA CAA CAG ACA AAT CA-3′ and antisense 5′-GGG GTG ATG TGT TTG AAC TTG-3′ for PPARγ; sense 5′-ATA TGA TAT GTC AGG TGC CCG-3′ and antisense 5′-CTT TGG TGA CAC ACA GTA TTA G-3′ for CASP14; sense 5′-ACA ACT TTG GTA TCG TGG AAG G-3′ and antisense 5′-GCC ATC ACG CCA CAG TTT C-3′ for GAPDH. Amplification was performed for 45 cycles. Each cycle consisted of denaturation at 95°C for 30 s, annealing at 55°C for 30 s, and extension at 72°C for 20 s. The mRNA levels were determined by calculating 2^−∆∆Ct^ values.

### Western Blot Analysis

The relative amount of filaggrin and filaggrin-2, GATA3, PPARα, PPARβ, PPARγ, and CASP14 proteins, regulated under the treatment of EBN samples, were quantified with specific antibodies. HaCaT keratinocytes were grown in a 6-well culture plate in concentration of 3 × 10^5^ cells/mL. After EBN treatment, the cells were lysed with low salt lysis buffer containing 150 mM NaCl [Affymetrix USB (part of Thermo Fisher Scientific), catalog number: 21618-5KG], 10 mM HEPES (Sigma-Aldrich, catalog number: H4034-100G), 1 mM EDTA (GoldBio, St Louis, United States, catalog number: E-210-500), 1 mM EGTA (Sigma-Aldrich, catalog number: E4378-100G), 1% NP-40 (Sigma-Aldrich, catalog number: NP40S-100ML), 0.01% SDS (Sigma-Aldrich, catalog number: 62862-1KG), 0.1 M Tris-HCl (pH 7.6) [Affymetrix USB (part of Thermo Fisher Scientific), catalog number: 75825-5KG] and protease inhibitors [1:1,000 aprotonin (Sigma-Aldrich, catalog number: A1153-25MG), 1:1,000 leupeptin (Sigma-Aldrich, catalog number: L2884-25MG), 1:200 benzamidine (Sigma-Aldrich, catalog number: 434760-5G), 1:1,000 pepstatin A (Sigma-Aldrich, catalog number: P5318-25MG)]. The protein concentration was measured by Bradford assay (Bio-Rad Laboratories). In the investigation of the p38-MAPK and GATA-3 phosphorylation, HaCaT keratinocytes were grown in a 12-well culture plate in concentration of 3 × 10^5^ cells/mL and stimulated with EBN samples at different time points with or without 2-h pre-incubation of p38 inhibitor SB203580 (Cell Signaling Technology, Danvers, United States, catalog number: 5633). D-sorbitol (S6021-500G) at 400 mM was applied as a positive control in p38 phosphorylation. The cells were lysed, and the lysates were dissolved in lysis buffer containing 0.125 M Tris-HCl, pH 6.8, 4% SDS, 20% glycerol [ACROS Organics (part of Thermo Fisher Scientific), catalog number: AC158922500], 2% 2-mercaptoethanol (Sigma-Aldrich, catalog number: M6250-100ML) and 0.02% bromophenol blue (Sigma-Aldrich, catalog number: 32712-5G) and denatured at 95°C for 5 min, three times. Reduced samples were normalized to 40 µg per lane and separated in 10% sodium dodecyl sulphate (SDS)-polyacrylamide gels. SDS-PAGE was run at 60–85 V and then transferred to nitrocellulose membranes. The membrane was blocked with 5% reduced fat milk or 5% BSA for 1 h at room temperature. After blocking, the membrane was incubated with primary antibodies overnight at 4°C. Primary antibodies used: mouse and rabbit anti-filaggrin antibody at 1:100 (Santa Cruz Biotechnology, Dallas, United States, catalog number: sc-66192 and sc-30229), rabbit anti-filaggrin-2 antibody at 1:1,000 (Bethyl Laboratories, Montgomery, United States, catalog number: A305-861A-M), rabbit anti-p38 MAPK antibody at 1:1,000 (Cell Signaling Technology, Danvers, United States, catalog number: 8690S), rabbit anti-phospho-p38 MAPK (Thr180/Tyr182) antibody at 1:1,000 (Cell Signaling Technology, catalog number: 4511S), rabbit anti-GATA3 antibody at 1:1,000 (Cell Signaling Technology, catalog number: 5852S), rabbit anti-phospho-GATA3 (Ser308) antibody at 1:1,000 (Thermo Fisher Scientific, catalog number: MA5-32144), rabbit anti-PPARα + PPARβ antibody at 1:2,000 (Abcam Ltd., Cambridge, United Kingdom, catalog number: ab1788865), rabbit anti-PPARγ antibody at 1:1,000 (Cell Signaling Technology, catalog number: 2435S), rabbit anti-CASP14 antibody at 1:1,000 (Cell Signaling Technology, catalog number: 8519S), and mouse anti-α-tubulin antibody at 1:10,000 (Sigma-Aldrich, catalog number: 3873S). The membranes were followed by secondary antibodies incubation for 2 h at room temperature, where HRP-conjugated antibodies at 1:2,000 (Zymed, South San Francisco, United States, catalog number: Rb7074S and Ms7076S) were used. Non-specific binding of protein was reduced with 0.1% Tween-20 TBST (pH 7.6) (anatrance, Maumee, United States, catalog number: T1003-500ML). The enhanced chemiluminescence (ECL) western blotting detection kit (Thermo Fisher Scientific, peroxide solution, catalog number: 1859701, luminol enhancer solution, catalog number: 1859698) was used. The protein amount was compared with the band intensities, measured under ChemiDoc Imaging System (Bio-Rad Laboratories).

### Frozen *ex vivo* Mouse Skin Section

The dorsal skin was shaved and collected from C57BL/6 mice (2-months old) after being sacrificed by cervical dislocation. The isolated skin was rinsed with PBS and cultured in DMEM supplemented with 10% (v/v) FBS and 1% (v/v) penicillin/streptomycin (10,000 U and 10,000 μg/mL) in a humidified atmosphere with 5% CO_2_ at 37°C. After the treatment, skin was rolled and embedded in optimal cutting temperature (OCT) compound (Thermo Fisher Scientific, catalog number: 6769006) in a tissue mold, followed by an overnight freezing under −80°C. Skin was then cut into 10 µm thick sections at −20°C using CryoStar™ NX70 Crystat (Thermo Fisher Scientific). The sections were melted onto slides, fixed with 4% paraformaldehyde (Sigma-Aldrich, catalog number: P6148-500G) for 30 min at room temperature and preserved at −80°C for following experiments.

### Hematoxylin and Eosin Staining

After sectioning, *ex vivo* mouse dorsal skin sections were fixed with 4% paraformaldehyde for 30 min at room temperature. The fixed skin sections were then stained with the hematoxylin and eosin (H and E) staining kit (catalog number: ab245880) purchased from Abcam, Ltd. Sections were first washed with PBS twice for 5 min and dried, before the incubation in hematoxylin solution for 5 min. Hematoxylin-stained sections were washed with DDI water twice. Incubation with bluing reagent was performed for 10–15 s, sections were then washed with DDI water twice followed by absolute ethanol. Incubation with eosin solution was subsequently performed for 3 s, and dehydration with absolute ethanol were conducted. Sample was then mounted with dibutyl phthalate in xylene (DPX) reagent (Sigma-Aldrich, catalog number: 06522-100ML) for microscopic evaluation. Samples were examined by a Zeiss Axio Vert. A1 inverted phase microscope (Zeiss, Jena, Germany) with a 10X objective.

### Immunofluorescent Staining

HaCaT keratinocytes were seeded on sterile coverslip (Marienfeld Superior, Lauda-Königshofen, Germany) in 35-mm culture plates at 5 × 10^4^ cells/mL. Cultured keratinocytes and *ex vivo* mouse dorsal skin sections were fixed with 4% paraformaldehyde for 30 min at room temperature, followed by blocking with 5% BSA in PBS with or without permeabilization by 0.1% triton for 2 h at room temperature. After blocking, the cell or tissue sample was incubated with primary antibodies, anti-FLG at 1:50 (Santa Cruz Biotechnology), anti-GATA3 at 1:100 or anti-PPARγ at 1:100, overnight at 4°C. The samples were followed by secondary antibodies incubation for 2 h at room temperature under darkness, where Alexa 488 (donkey anti-mouse IgG, catalog number: ab150105) and 647 (donkey anti-rabbit IgG, catalog number: ab150075)-conjugated antibodies at 1:200 (Abcam Ltd.) were used. Samples were mounted with ProLong Gold Antifade Mountant with DAPI (Thermo Fisher Scientific, catalog number: P36931). Samples were then examined by a Leica SP8 Confocal Microscope (Leica Microsystems, Wetzlar, Germany) with a 63X oil immersion objective.

### Atomic Force Microscopy

The mechanical properties of HaCaT keratinocytes were characterized by atomic force microscope (AFM) (MFP-3D, Asylum Research, Santa Barbara, United States) supplemented with a colloidal probe. The colloidal probe was prepared by gluing a glass sphere (∼12 μm in diameter) onto the free end of a tipless cantilever (CSC38, tipless, MikroMasch) with a typical spring constant k∼0.09N/m (Shen et al., 2017). The exact spring constant of the modified cantilever was calibrated by thermal power spectral density method (Shen et al., 2017). Before each AFM experiment, the probe surface was coated with a thin layer of poly (l-lysine)-graft-poly (ethylene glycol) (PLL-g-PEG) (SuSoS AG, Dübendorf, Switzerland) to avoid adhesion between the probe and cell surface. Cultured keratinocytes for AFM measurement were seeded onto a sterile coverslip (Marienfeld Superior) in 35-mm culture plates at 5 × 10^4^ cells/mL and cultured in DMEM supplemented with 10% (v/v) FBS and 1% (v/v) penicillin/streptomycin (10,000 U and 10,000 μg/mL). After the application of EBN samples, including extract and digest, for 24 h, cells were rinsed with sterile PBS twice and then transferred into an AFM closed fluid chamber, where ambient buffer environment was provided. The AFM measurement for EBN-treated cells was performed in PBS solution at 37°C in a humidified atmosphere of 5% CO_2_. AFM measurements were also performed in hypertonic and hypotonic PBS solutions to characterize different cell properties in various osmotic conditions, serving as negative and positive controls. The hypotonic and hypertonic PBS solutions were prepared based on Ayee et al. (2018), with osmolarity of 227 and 366 mOsm/kg, respectively, measured by Advanced Model 3320 Micro-Osmometer (Advanced Instruments, Norwood, United States).

### Statistical Analysis

Data for control and drug-treated groups were compared using one-way ANOVA with Dunnett post-hoc statistical testing provided in GraphPad Prism 8.3.0. Results were calculated from at least 3 independent determinations, performed in triplicates and expressed as percent increase or fold change of control in mean ± SEM. Statistical significance was indicated by * *p* < 0.05, ** *p* < 0.01, and *** *p* < 0.001. Statistical comparison in Figure 8B (lower panel) was made by two tailed *t*-test, * *p* < 0.05, and *** *p* < 0.001, compared to indicated group pre-treated with SB203580.

## Results

### The Extract and Digest of EBN Induce Expressions of Filaggrin and Filaggrin-2

**FIGURE 1:**
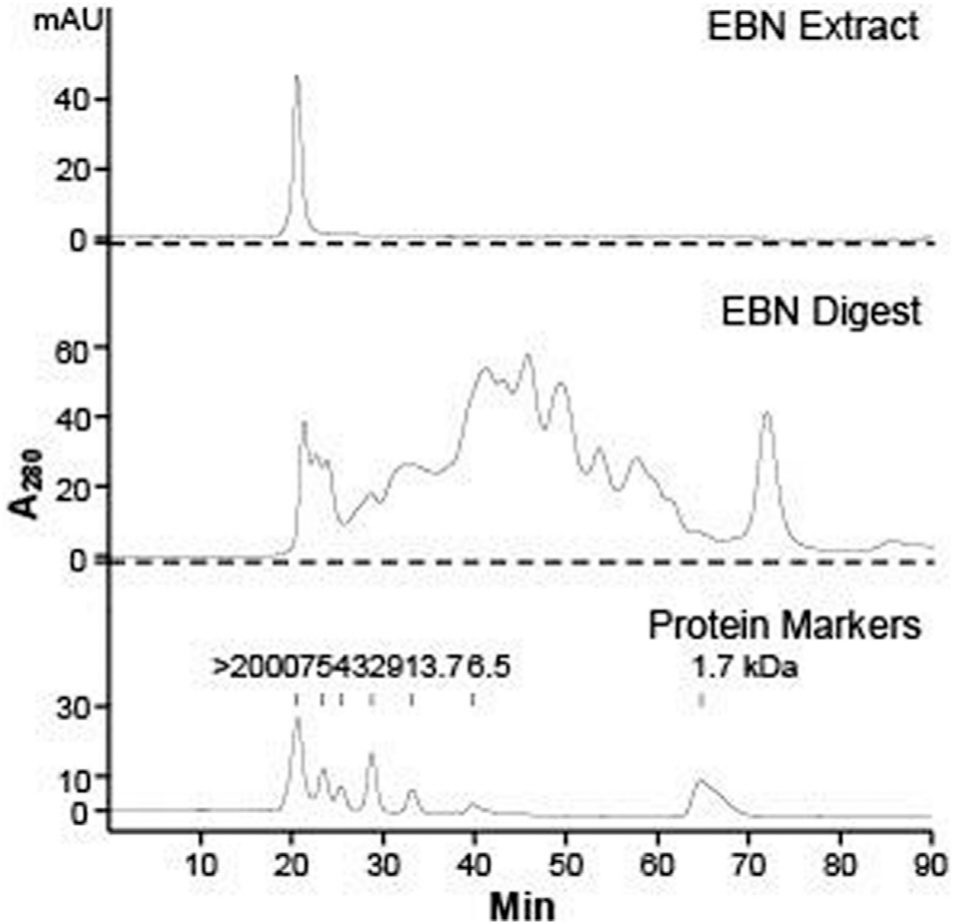
HPLC chromatogram of EBN extract and digest. Gel chromatographic separations of Malaysia white house EBN extract (10 mg/mL, injection volume = 10 μL) and digest (10 mg/mL, injection volume = 100 μL) under UV wavelength of 280 nm are shown. The molecular weight of protein markers of α-melanocyte-stimulating hormone (1.7 kDa), aprotonin (6.5 kDa), ribonuclease A (13.7 kDa), carbonic anhydrase (29 kDa), ovalbumin (43 kDa), albumin (75 kDa), and blue dextran (> 2000 kDa) are indicated.

The HPLC chromatograms of EBN extract and its enzymatic digested product were illustrated in Figure 1, which were obtained by injections of 10 µL of 10 mg/mL EBN extract, or 100 µL 10 mg/mL EBN digest, or 10 µL 1 mg/mL protein markers. The UV absorbance of protein/peptide was measured at wavelength 280 nm. The peak eluted at 20 min was the only peak observed from EBN extract, suggesting that EBN was formed in a large complex protein with an oversized molecular weight at least >2,000 kDa. After 48 h of digestion by simulated gastric fluid with pepsin, the digested product was subjected to HPLC analysis. In the EBN digest, numerous peaks were identified in the chromatogram, and the peak at 20 min of undigested EBN complex was markedly decreased, i.e. digestion to smaller protein/peptide (Figure 1). The chromatogram of protein markers suggested that most of the digested EBN was migrated at small molecular weight, i.e. ∼1.7 kDa. By estimation, the protein digestion reached over 90% of completion. This fingerprint of digested EBN could serve as a quality control of the product.

**FIGURE 2:**
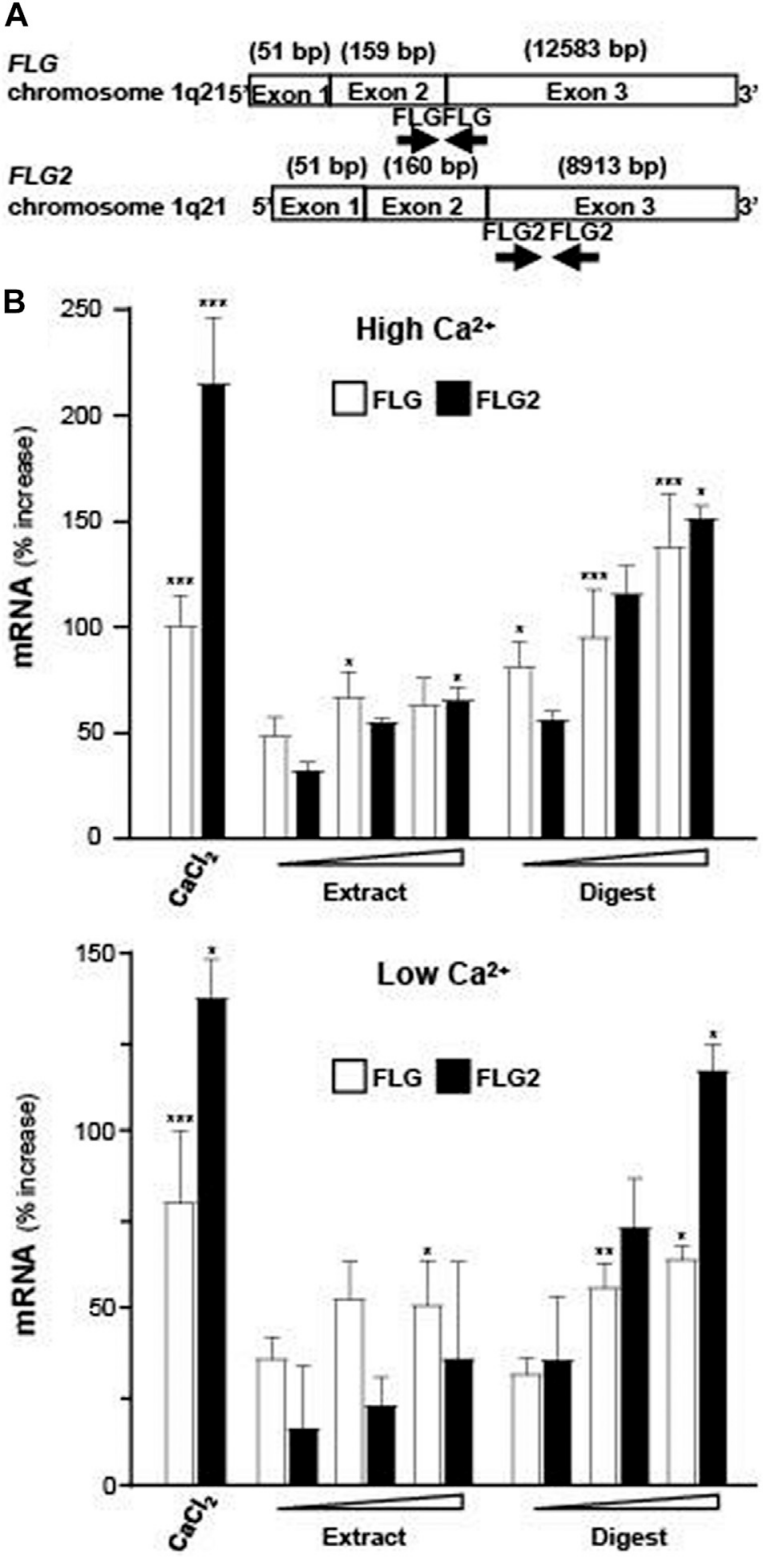
Expressions of filaggrin and filaggrin-2 are evaluated by real-time PCR. **(A)** The schematic diagrams of primers flanking the exons of human filaggrin (FLG) and filaggrin-2 (FLG2) genes on chromosome 1q21 are indicated. **(B)** Filaggrin and filaggrin-2 mRNA levels in cultured differentiated (upper panel; 1.8 mM Ca^2+^) and undifferentiated (lower panel; low Ca^2+^) keratinocytes after 24-h treatments of EBN extract and digest, in doses of 1, 10, 100 μg/mL, as indicated. CaCl_2_ at 0.16 mM was adopted as a positive control. Values are expressed as the percentage of increase in relative to normalized basal expression set at 0, in mean ± SEM, *n* = 4. Statistically significant results are marked with * *p* < 0.05, ** *p* < 0.01, and *** *p* < 0.001 against the control.

To assess the ability of EBN in promoting the moisturizing effect of skin keratinocytes, the extract and digest of EBN were tested for regulation of mRNAs encoding filaggrin and filaggrin-2, two specific proteins playing critical role in skin water maintenance. Figure 2A shows the location of primers flanking the two genes. The EBN digest, instead of EBN extract, showed robust induction of filaggrin and filaggrin-2 expressions in dose-dependent manners: the maximal induction was at over 2.5-fold as compared to the control under high calcium condition (1.8 mM) (Figure 2B upper panel). This induction was also revealed in low calcium culture medium, where EBN digest again showed more significant induction, peaked at 1.5 to 2.2-fold of control (Figure 2B lower panel). The mRNA inductions of filaggrin and filaggrin-2 were higher under culture condition with higher Ca^2+^ concentration. In both scenarios, the EBN digest showed much better efficacy in inducing the mRNA expression than that of EBN extract, suggesting the digestion of EBN could generate effective peptides and/or small chemicals. External applied Ca^2+^ was adopted as a positive control (Li et al., 1995).

**FIGURE 3:**
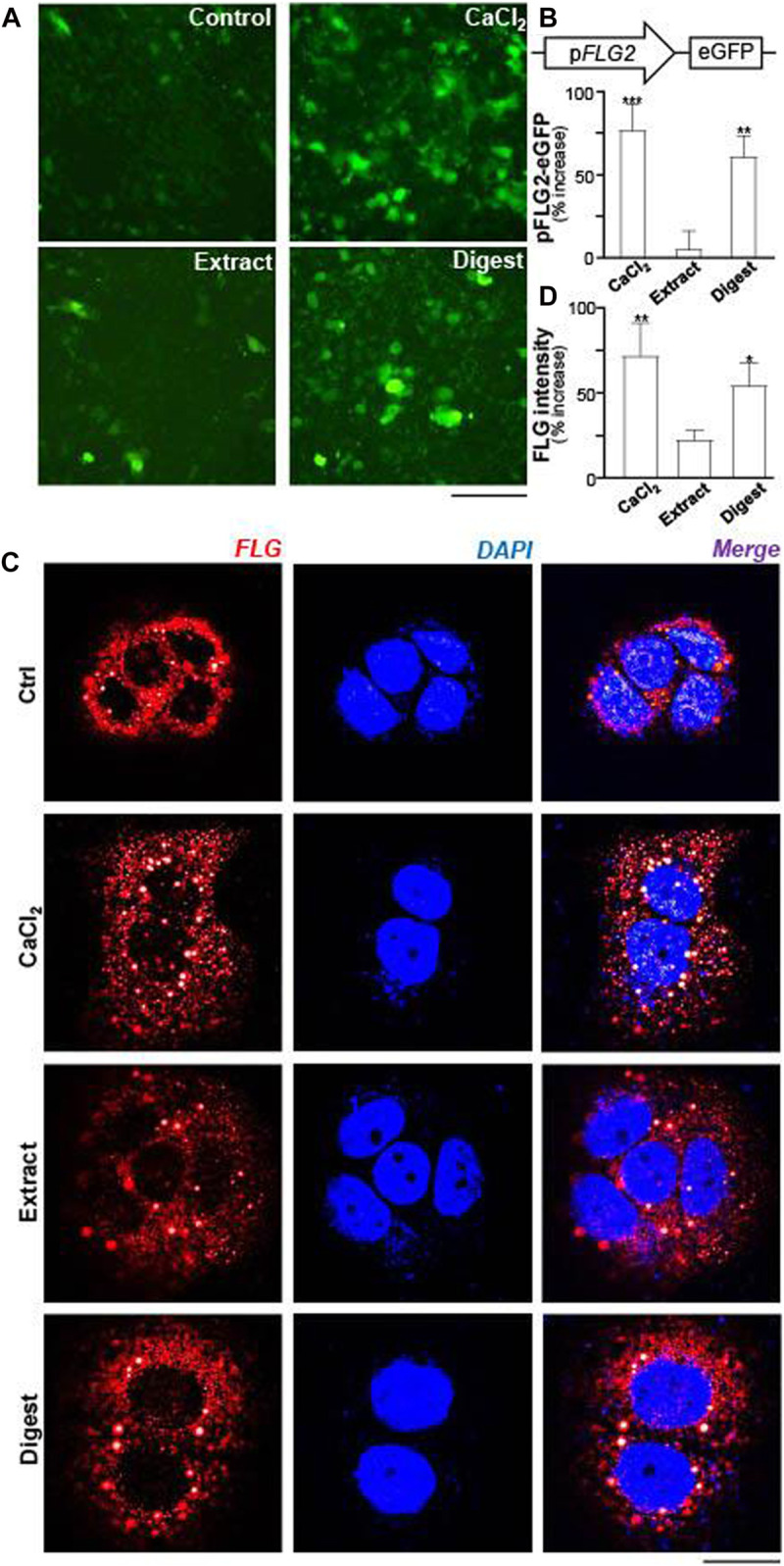
EBN induces the activity of pFLG2-eGFP and filaggrin expression. **(A)** The fluorescence images of pFLG2-eGFP transfected keratinocytes after 24-h treatments of CaCl_2_ (0.16 mM, a positive control), EBN extract (100 μg/mL), and EBN digest (100 μg/mL) are shown, *n* = 3. Bar = 100 μm. The pFLG2-eGFP plasmid was composed of FLG2 promoter tagged with EGFP reporter. **(B)** The quantifications from **(A)** are expressed as the percentage of increase in fluorescence intensity measured under excitation/emission wavelength 488/509 nm to normalized basal activity set at 0 in mean ± SEM, *n* = 6. (**C)** Cytosolic expression of FLG protein in HaCaT keratinocytes after 24-h treatments of EBN extract (100 μg/mL) and EBN digest (100 μg/mL) are shown, in representative confocal images. CaCl_2_ at 0.16 mM was adopted as a positive control. *n* = 3. Bar = 20 μm. **(D)** The quantifications from **(C)** are expressed as the percentage increase in filaggrin fluorescence intensity to normalized basal activity set at 0 in mean ± SEM, *n* = 3. The filaggrin fluorescence intensity of each cell was normalized to total cell area for quantification. Statistically significant results are marked with * *p* < 0.05, ** *p* < 0.01, and *** *p* < 0.001 against the control.

The promoters of filaggrin-2, tagged downstream with an eGFP construct (pFLG2-eGFP), was used here for illustration of activated gene transcription. In pFLG2-eGFP transfected keratinocytes, the fluorescent signal, representing the promoter activity, was induced by applications of EBN extract and EBN digest (Figure 3A). The quantitation showed robust activity, at least 50% increase of pFLG2-eGFP fluorescence, triggered by EBN digest, similar to the positive control of Ca^2+^ application: the extract of EBN showed insignificant effect (Figure 3B). Filaggrin is a non-membrane-bound protein localized in keratohyalin granules (Presland et al., 2001). Thus, the amount of filaggrin protein was revealed by immunochemical staining in cultured keratinocyte. The treatments of EBN extract and digest in keratinocyte induced the overall cellular staining of filaggrin, which again showed better effect in the scenario of EBN digest (Figures 3C,D).

**FIGURE 4:**
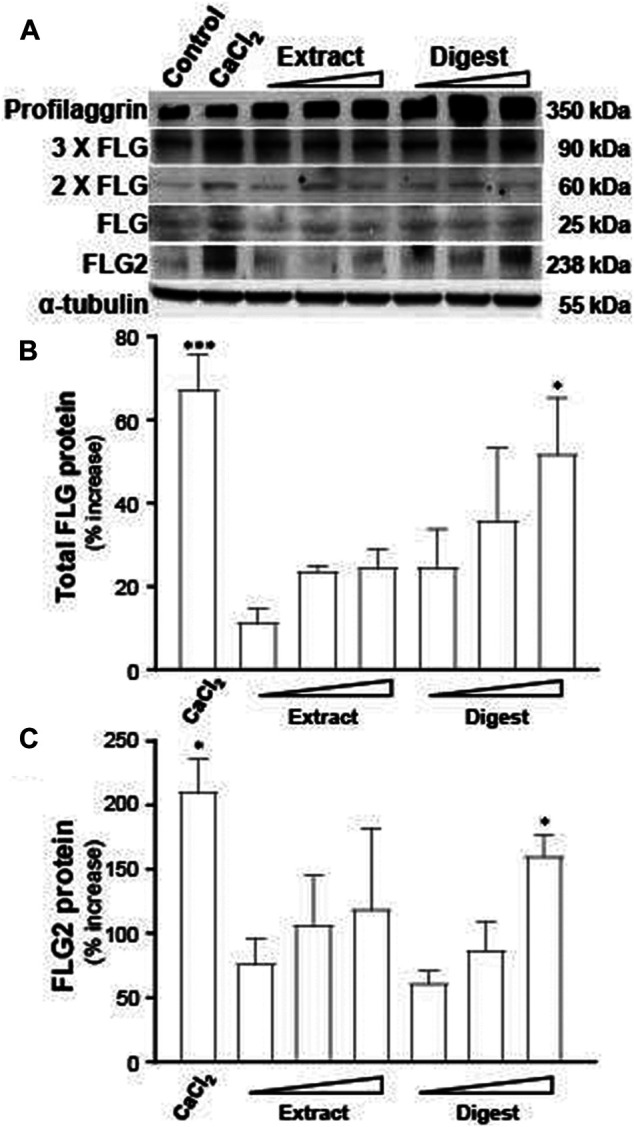
EBN induces the protein levels of filaggrin and filaggrin-2. **(A)** The protein levels of filaggrin (FLG) and filaggrin-2 (FLG2) in cultured differentiated keratinocytes, after 24-h treatments of EBN extract and digest, were determined in doses of 1, 10, 100 μg/mL, as indicated. CaCl_2_ at 0.16 mM was adopted as a positive control. **(B)** The total filaggrin (summation of the isomeric intermediates) and **(C)** filaggrin-2 protein levels, relative to α-tubulin protein, are expressed as the percentage increase to normalized basal expression set at 0 in mean ± SEM, *n* = 3. Statistically significant results are marked with * *p* < 0.05, ** *p* < 0.01, and *** *p* < 0.001 against the control.

The protein expressions of filaggrin and filaggrin-2, induced by EBN, were determined by western blotting. Profilaggrin is dephosphorylated, deiminated and proteolyzed into isomeric repeat intermediates. The bands at ∼350 kDa referred to profilaggrin, showing the multimeric complex (Figure 4A). The total filaggrin expression was summed for the band intensities corresponding to trimeric (∼90 kDa), dimeric (∼60 kDa), and monomeric (∼25 kDa) forms in the blot (Figure 4A). In parallel, the band of ∼238 kDa was corresponding to flaggrin-2 (Figure 4A). By quantitation of the band intensities, the digest of EBN showed induction of protein expressions, both in filaggrin (Figure 4B) and filaggrin-2 (Figure 4C), significantly, which were in dose-dependent manners. EBN extract showed the protein induction at low magnitude than that of the digest, which was in line to the results of mRNA and promoter assays.

### Signaling of EBN-Induced Expression of Filaggrin and Filaggrin-2

**FIGURE 5:**
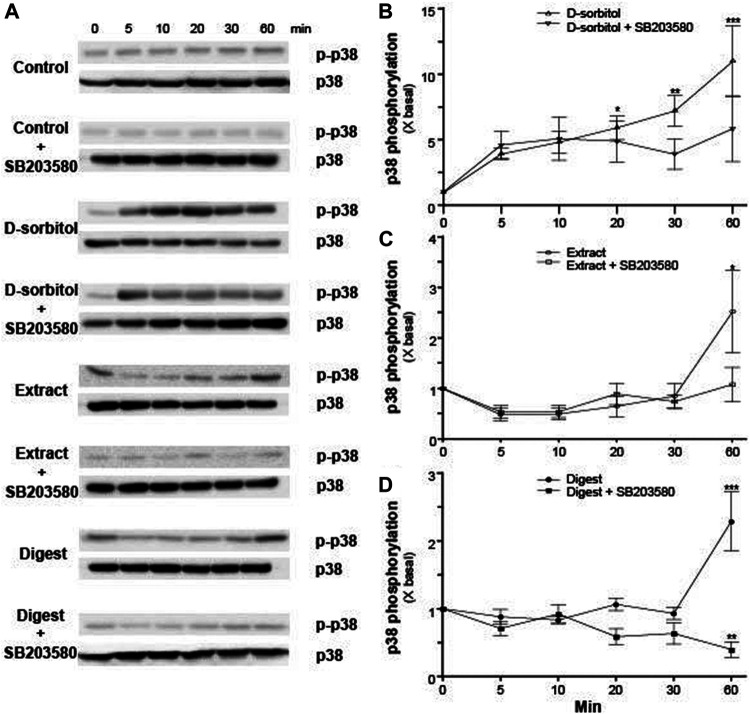
The p38 MAPK inhibitor blocks EBN-induced p38-MAPK phosphorylation. **(A)** After 16-h serum starvation, cultured differentiated keratinocytes were pre-incubated with or without SB203580 (a p38 kinase inhibitor; 10 μM) for 2 h, followed by different times of applied D-sorbitol (a positive control, 400 mM), EBN extract or digest (both at 100 μg/mL). Total p38-MAPK (p38-MAPK) and phosphorylated p38-MAPK (p-p38 MAPK) (both at ∼38 kDa) were evaluated by western blot assays. The quantifications of p-p38 MAPK/p38 MAPK expression levels after different time of applied **(B)** D-sorbitol, **(C)** EBN extract, and **(D)** EBN digest with or without SB203580 pretreatment are shown. Values are expressed in mean ± SEM, *n* = 3. Statistically significant results are marked with * *p* < 0.05, ** *p* < 0.01, and *** *p* < 0.001 against the control.

MAPK signaling pathways have been shown to mediate the functions of keratinocyte differentiation and skin barrier (Meng et al., 2018). The activation of p38 resulted from the phosphorylation of Thr-Gly-Tyr (TGY) motif in kinase domain (Cargnello and Roux, 2011). To identify whether EBN in regulating the expressions of filaggrin and filaggrin-2 *via* p38-MAPK signaling, the p38-MAPK phosphorylation, activated by EBN extract and digest, was performed. The treatment of EBN extract and digest increased the p-p38 MAPK/p38 MAPK ratio by 2–3 folds after 60 min (Figures 5A,C,D). Furthermore, the pre-incubation of p38 MAPK inhibiter SB203580 significantly suppressed the activation of p38-MAPK, as triggered by EBN extract and/or digest (Figures 5A,C,D), showing the specificity of EBN-activated p38-MAPK pathway. D-sorbitol served as a positive control in activating p38 phosphorylation (Clerk et al., 1998) (Figures 5A,B).

**FIGURE 6:**
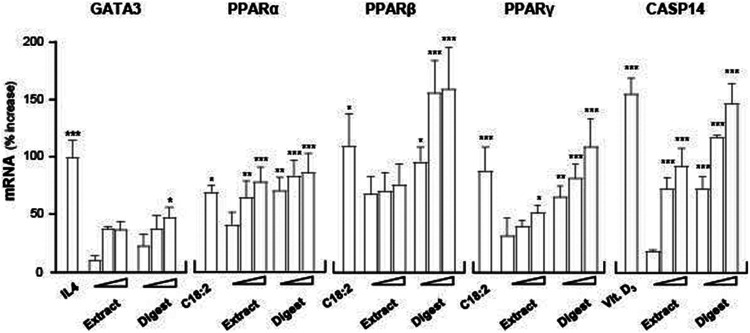
EBN induces mRNA expressions of targeted genes corresponding to moisturize skin. The mRNA levels of GATA3, PPARα, PPARβ, PPARγ, and CASP14 in cultured differentiated keratinocytes after 24-h treatments of EBN extract and digest were determined in doses of 1, 10, 100 μg/mL (as indicated). IL-4 at 100 ng/mL, linoleic acid (C18:2) at 30 μM and cholecalciferol (vitamin D_3_) at 10^–5^ M were adopted as positive controls in GATA3, PPARs, and CASP14 expressions. Values are expressed as the percentage of increase in mRNA amount relative to normalized basal expression set at 0 in mean ± SEM, *n* = 4. Statistically significant results are marked with * *p* < 0.05, ** *p* < 0.01, and *** *p* < 0.001 against the control.

GATA3, a key transcriptional factor in regulating the expressions of filaggrin and filaggrin-2, was hypothesized here to be involved as an upstream target of the EBN-mediated gene expression in cultured keratinocytes. To validate the role of GATA3, RT-PCR was employed to quantify the expression of GATA3 mRNA under the treatment of EBN. The treatment of EBN digest increased the mRNA level by ∼50% in a dose-dependent manner, better than that of EBN extract (Figure 6). IL-4 served as a positive control in activating GATA3 transcription (Onderdijk et al., 2015).

Peroxisome proliferator activated receptors (PPARs) are ligand-activated nuclear transcription factors expressed in human skin keratinocytes, and the reported subtypes were PPARα, PPARβ, and PPARγ (Sertznig et al., 2008). The three PPAR variants are known to induce filaggrin expression in both *in vitro* and *in vivo* models (Harding and Rawlings, 2000). To evaluate whether PPARs participated as an upstream target in the EBN-mediated regulation of filaggrin and filaggrin-2 in cultured keratinocytes, the mRNA levels of PPARα, PPARβ, and PPARγ were determined. Linoleic acid (C18:2), a positive control (Kliewer et al., 1994), activated the PPAR variants by 50–150% of increase (Figure 6). In cultured keratinocytes treated with EBN, the amounts of PPARα, PPARβ, and PPARγ mRNAs were increased in dose-dependent manners (Figure 6). The inducing response was more robust in a scenario of PPARβ under the challenges of EBN digest. In all scenarios, the extract of EBN showed much less induction as that of EBN digest.

Caspase 14 (CASP14) is a Ca^2+^-dependent cysteine protease involved in the cell senescence of epidermal keratinocytes. CASP14 plays roles in proteolysis and deimination of profilaggrin and filaggrin-2 proteins, leading to keratinocyte differentiation and cornified envelope formation (Hoste et al., 2011; Masse et al., 2014). To assess the ability of EBN in upregulating CASP14 expression, RT-PCR assay was performed. 1α, 25-dihydroxycholecalciferol (vitamin D_3_) was adopted as a positive control (Lippens et al., 2004). EBN extract and digest increased the expression levels of CASP14 in a dose-dependent manner: the EBN digest showed better response (Figure 6). Thus, EBN could be able to induce CASP14 expression involving in the proteolysis of filaggrin and filaggrin-2 to form NMFs during late differentiation of keratinocyte.

**FIGURE 7:**
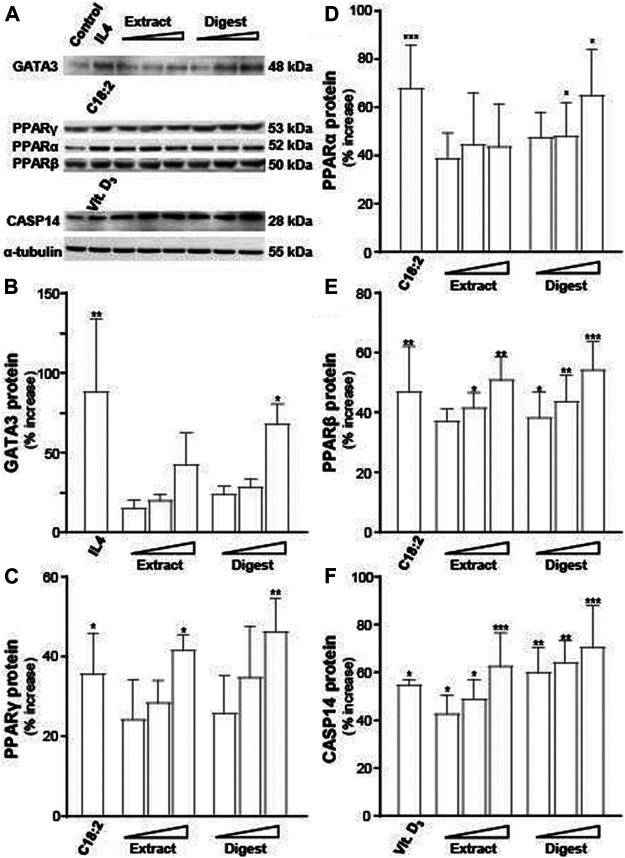
EBN induces the expressions of targeted proteins corresponding to moisturize skin. **(A)** Western blot membranes showing GATA3, PPARs, and CASP14 protein expressions after 24-h treatments of EBN extract and digest were assessed in doses of 1, 10, 100 μg/mL (as indicated). IL-4 at 100 ng/mL, linoleic acid (C18:2) at 30 μM and cholecalciferol (vitamin D_3_) at 10^–5^ M were adopted as positive controls in GATA3, PPARs, and CASP14 expressions. The quantification of **(B)** GATA3, **(C)** PPARγ, **(D)** PPARα, **(E)** PPARβ, and **(F)** CASP14 are shown. The expression levels, in relative to α-tubulin protein, are expressed as the percentage of increase to normalized basal expression set at 0 in mean ± SEM, *n* = 3. Statistically significant results are marked with * *p* < 0.05, ** *p* < 0.01, and *** *p* < 0.001 against the control.

Besides mRNA, the protein expressions of GATA3 (∼48 kDa), PPARα (∼53 kDa), PPARβ (∼50 kDa), PPARγ (∼53 kDa), and CASP14 (∼28 kDa) were determined (Figure 7A). α-Tubulin served as a loading control. In all scenarios, the treatments of EBN extract and digest in cultured keratinocytes were able to significantly induce the protein expressions, in dose-dependent manners (Figures 7B–F). The robust activation, triggered by EBN digest, was revealed in all cases (Figure 7). EBN extract showed protein induction as well but at a lower magnitude to that of EBN digest.

**FIGURE 8:**
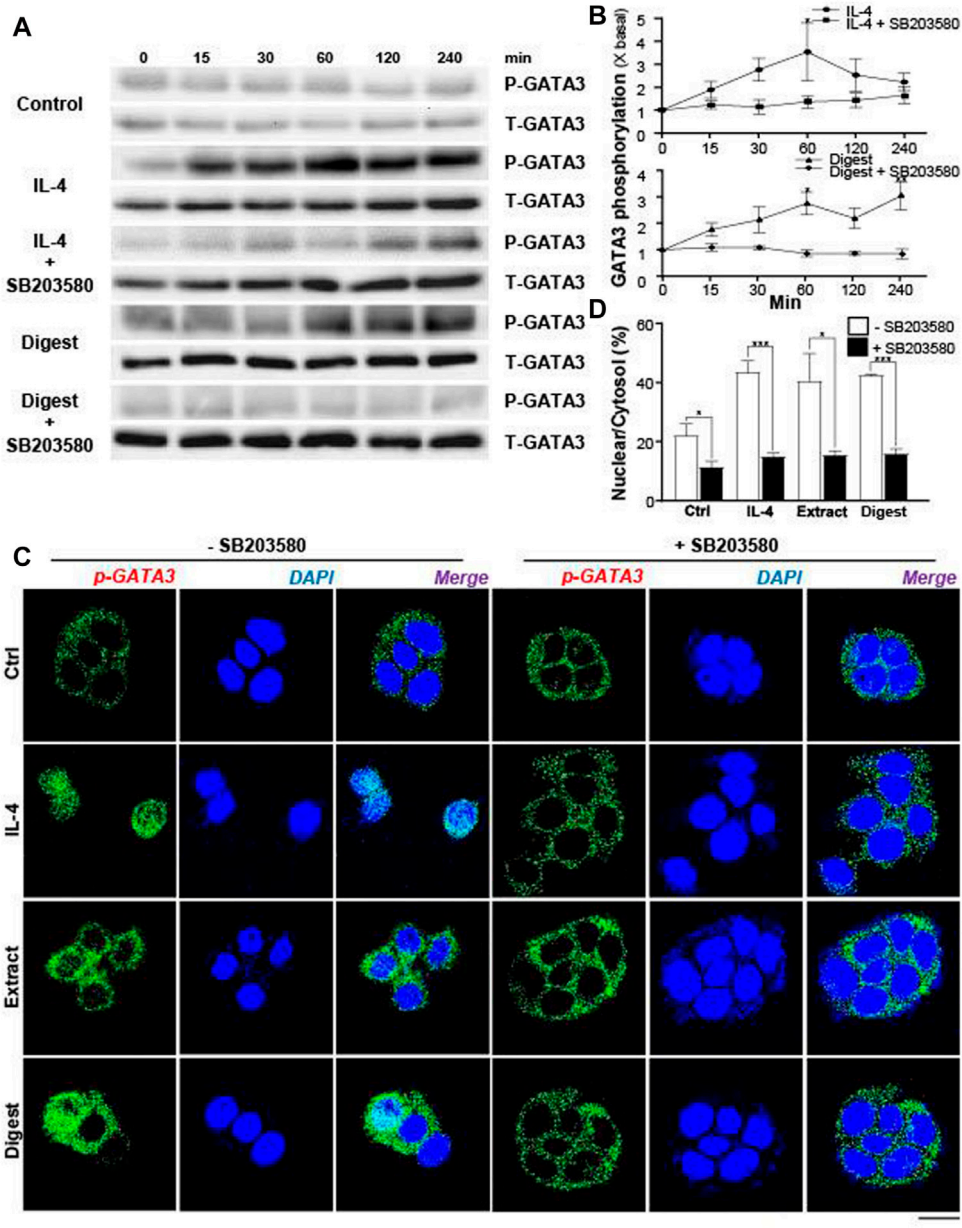
EBN induces phosphorylation of GATA3 and its nuclear translocation. **(A)** After 16-h serum starvation, cultured differentiated keratinocytes were pre-incubated with or without SB203580 (10 μM) for 2 h, followed by different time of applied IL-4 (a positive control, 100 ng/mL) or EBN digest (100 μg/mL). Total GATA3 (T-GATA3) and phosphorylated GATA3 (P-GATA3) (both at ∼48 kDa) were evaluated by western blot assays. **(B)** The quantification of the increased fold of P-GATA3/T-GATA3 expression levels from **(A)** after different time of applied IL-4 and EBN digest, with or without SB203580 pre-treatment, are shown. Values are expressed in mean ± SEM, *n* = 3. Statistically significant results are marked with * *p* < 0.05, ** *p* < 0.01, and *** *p* < 0.001 against the control. **(C)** After 16-h serum starvation, cultured differentiated keratinocytes were pre-incubated with or without SB203580 (10 μM) for 2 h, followed by 240-min treatment of IL-4 (100 ng/mL, a positive control), EBN extract (100 μg/mL) and EBN digest (100 μg/mL). Phosphorylated GATA3 protein localization in keratinocytes was determined by immunofluorescence assays. *n* = 3. Bar = 20 μm. **(D)** The quantification of the percentage of nuclear to cytosol level of P-GATA3 intensities under different treatments from **(C)** are shown. Values are expressed in mean ± SEM, *n* = 3. Statistically significant results are marked with * *p* < 0.05, ** *p* < 0.01, and *** *p* < 0.001 against the control or as indicated.

The levels of those transcriptional factors are not crucial regulators to activate the expressions of flaggrin and/or flaggrin-2 in skin cell. Instead, the activation of transcriptional factors by phosphorylation or dimerization is the key in regulating the gene transcription. For example, the phosphorylation of *p*-GATA3 by ∼3-fold in keratinocytes was triggered by IL-4, a positive control, in a transient manner: the IL-4 induced phosphorylation was blocked by a p-38 MAPK inhibitor, i.e., SB203580 (Figures 8A,B). The digest of EBN activated the phosphorylation of GATA3 in a time-dependent manner to a maximal increase of ∼2.5-fold (Figures 8A,B). Similar to the situation of IL-4, the phosphorylation was blocked by applied SB203580.

**FIGURE 9:**
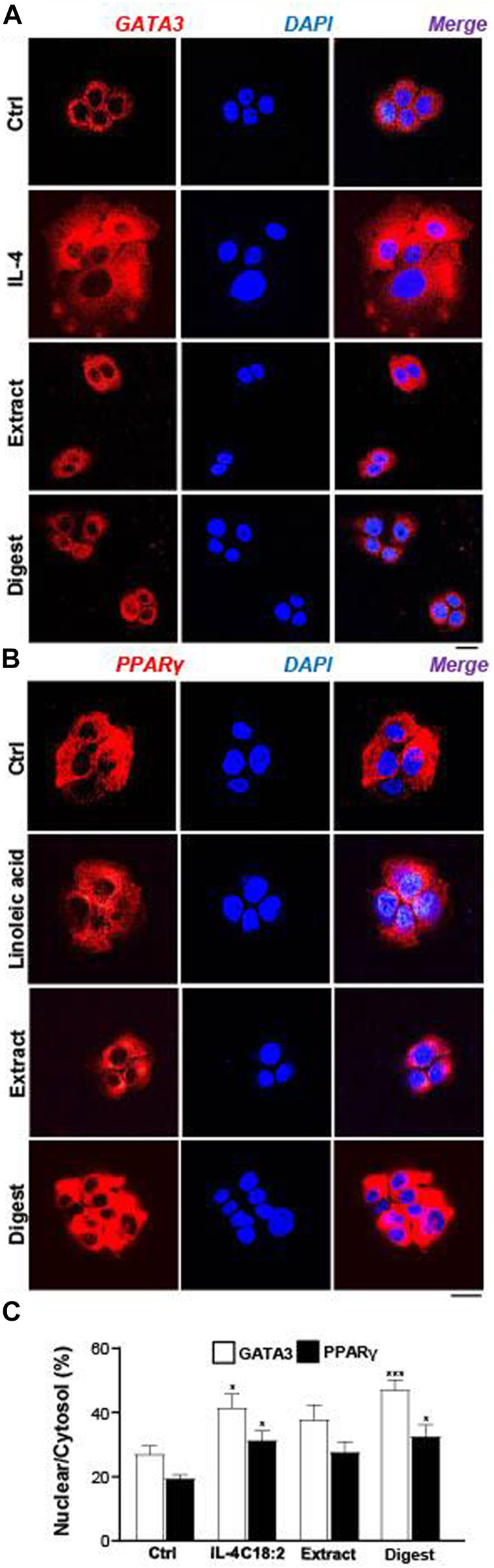
EBN induces nucleus localizations of GATA3 and PPARγ proteins. The immunofluorescent staining of **(A)** GATA3 or **(B)** PPARγ in cultured differentiated keratinocytes after 24-h treatments of EBN extract (100 μg/mL) and EBN digest (100 μg/mL) are shown. IL-4 at 100 ng/mL and linoleic acid (C18:2) at 30 μM were adopted as positive controls in GATA3 and PPARγ expression. Bar = 20 μm. **(C)** The quantification of the percentage of nuclear GATA3 to cytoplasmic GATA3, or PPARγ, are shown. Values are expressed in mean ± SEM, *n* = 3. Statistically significant results are marked with * *p* < 0.05, ** *p* < 0.01, and *** *p* < 0.001 against the control.

In EBN-treated keratinocyte, the translocation of *p*-GATA3 was identified by immunochemical staining. The application of EBN extract and digest induced the nucleus localization of *p*-GATA3, as revealed by specific staining at the nucleus (Figure 8C). In addition, the nuclear translocation of GATA3 was inhibited by the p-38 MAPK inhibitor (SB203580) (Figures 8C,D). In parallel, the translocation of *p*-GATA3 from cytosol to nucleus was further confirmed by staining with total GATA3 specific antibody, which had recognized the nuclear localization of the protein after IL-4 and EBN treatments (Figure 9A). In addition, the nucleus translocation of another transcriptional factor, PPARγ, was illustrated. Application of EBN extract/digest and linoleic acid (a positive control) in keratinocyte induced the accumulation of PPARγ in the nucleus (Figure 9B). By quantification of percentage of GATA3 and PPARγ intensities in nuclear to cytosol, the digest of EBN showed more significant activation and translocation of the transcription factors (Figure 9C).

### EBN Induces Filaggrin in Skin and Cell Moisturization in Keratinocyte

**FIGURE 10:**
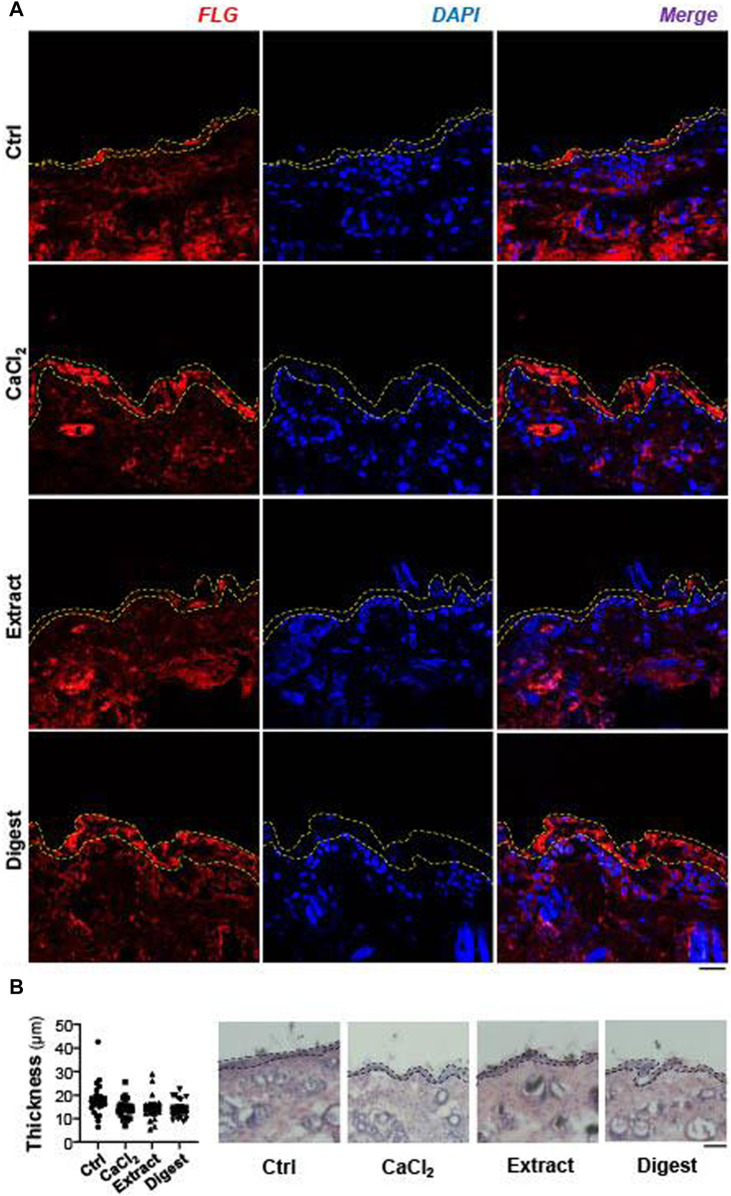
EBN induces filaggrin in *ex vivo* mouse skin. **(A)** The expression of filaggrin (FLG) protein in 2-month-old mouse dorsal skin *ex vivo* after 24-h treatments of EBN extract (100 μg/mL) and EBN digest (100 μg/mL) were assessed by immunofluorescent staining. CaCl_2_ at 0.16 mM was adopted as a positive control, *n* = 3. Bar = 20 μm. Yellow dash lines indicate the fluorescent area of filaggrin. **(B)** The epidermal thickness of *ex vivo* mouse dorsal skin after 24-h treatments of CaCl_2_ (0.16 mM), EBN extract (100 μg/mL) and EBN digest (100 μg/mL) were assessed by H and E staining. Black dash lines include epidermis layer (right panel). The quantification of five randomly selected distance points were assessed for the epidermal thickness (left panel). *n* = 3. Bar = 100 μm.

The functional role of EBN in regulating filaggrin and filaggrin-2 was further demonstrated in mouse skin *ex vivo*. After the treatment with EBN, the skin section was stained with filaggrin. The amount of staining was robustly increased in the epidermal level of skin, after treatment with EBN extract and digest, as well as the control of external applied Ca^2+^ (Figure 10A). All treatments did not increase the epidermal thickness (Figure 10B). Similar to the cell culture study, the effect of EBN digest showed more robust induction of filaggrin expression, i.e. moisturizing effect, as compared to EBN extract.

**FIGURE 11:**
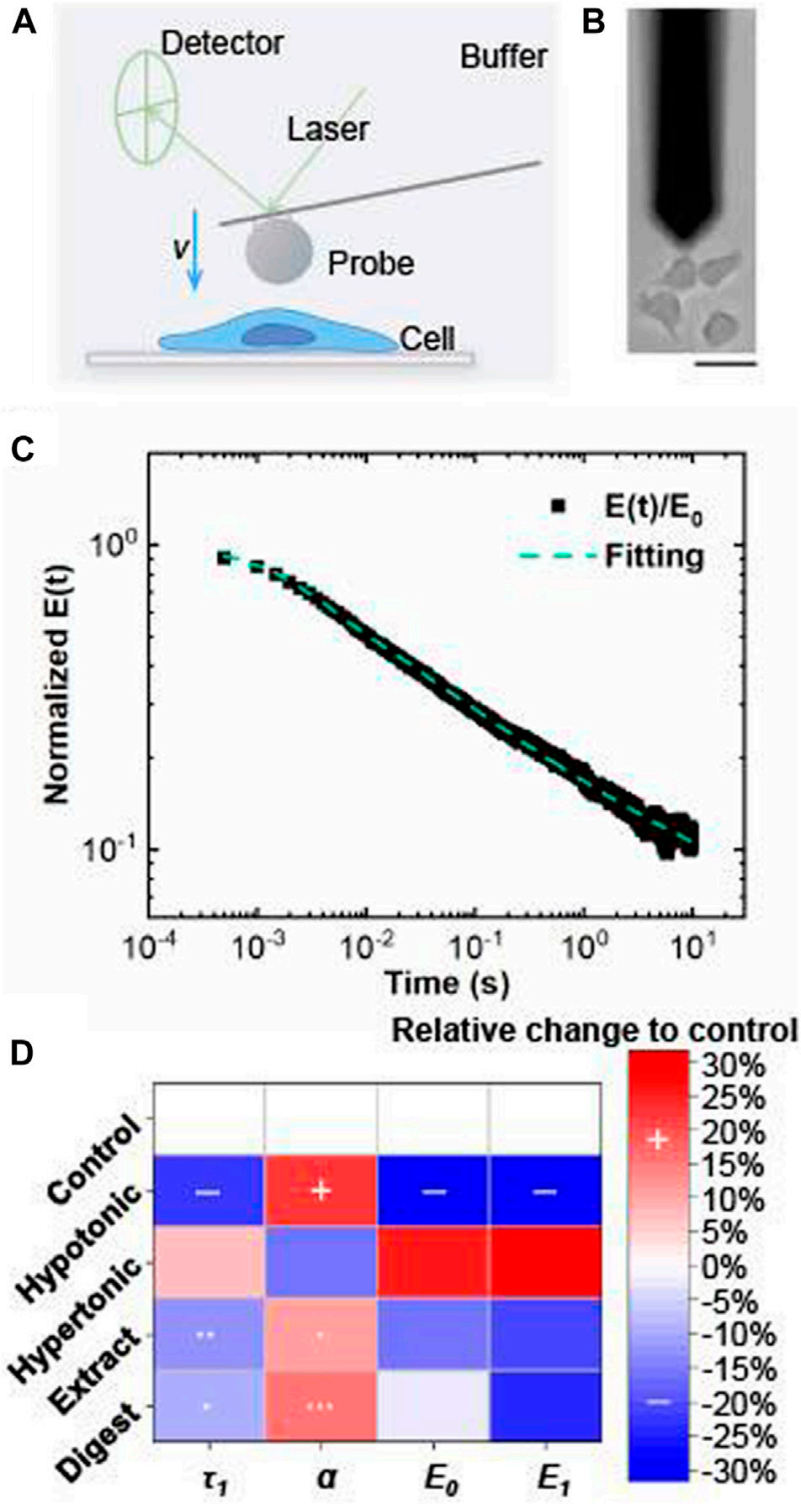
Viscoelastic properties of EBN-treated keratinocyte evaluated by atomic force microscopy. **(A)** Atomic force microscopy (AFM) setup for mechanical measurements of single cell (side view). **(B)** Bright field image of AFM measurement (top view). Bar = 35 μm. **(C)** A sample curve of normalized relaxation modulus $E\left(t\right)/E_{0}$ as the function of time. Green dash line is fitting to the measured relaxation data, showing a short-time exponential decay followed by a long-time power-law decay. **(D)** Relative changes of $E_{0}$, $E_{1}$, $\tau_{1}$, and α to the control group under different treatments in cultured keratinocyte, as in Figure 2. Simultaneous decrease of $E_{0}$, $E_{1}$, $\tau_{1}$, and increase of α show the moisturization of cells; while increase of $E_{0}$, $E_{1}$, $\tau_{1}$, and decrease of α show the dehydration of cells. The parameter map indicates that EBN-treated keratinocyte (100 μg/mL extract *n* = 30, 100 μg/mL digest *n* = 31) becomes moisturized, similar to cells under hypotonic condition (227 mOsm/kg, *n* = 6) and opposite to hypertonic one (366 mOsm/kg, *n* = 10). Statistically significant results are marked with * *p* < 0.05, ** *p* < 0.01, and *** *p* < 0.001 against the control.

With a multiscale relaxation model, the viscoelastic properties of cultured keratinocytes could be measured by AFM (Figures 11A,B) and described with relaxation modulus:

$$E\left(t\right)=E_{1}e^{-\frac{t}{\tau_{1}}}+E_{2}{\left(1+\frac{t}{\tau_{2}}\right)}^{-\alpha }+E_{\infty }$$

The relaxation modulus E(t) consists of three parts, namely a short-time exponential decay followed by a long-time power law decay, and a persistent modulus that does not vanish at an infinite time. In the above equation, $E_{1},E_{2},E_{\infty }$ are the moduli of exponential component, power-law component, and persistent component, respectively; $\tau_{1}$, $\tau_{2}$ are the scales for exponential decay and power-law decay; α is the power-law exponent. The initial modulus $E_{0}=E_{1}+E_{2}+E_{\infty }$ is the measured modulus at time t=0, which is considered as a measure of apparent rigidity of whole cell when indenting at high speed. A typical measurement of normalized relaxation modulus $E\left(t\right)/E_{0}$ is given (Figure 11C).

In this relaxation model, the four parameters: initial modulus $E_{0}$, exponential modulus component $E_{1}$, exponential relaxation time $\tau_{1}$ and power-law exponent α are jointly correlating with the moisturization states of the cells. The reasons could be shown below. Firstly, the short-time exponential relaxation represents the reorganization of biopolymers in cytosol. When the cell absorbs water due to osmotic stress or moisturization, the cell becomes softer, and therefore the measured modulus $E_{0},E_{1}$ become smaller (Moeendarbary et al., 2013; Liao et al., 2018). In addition, the exponential relaxation time is $\tau_{1}∼b^{2}/D$ (*b* is the mesh size of cytoskeleton network, and D is the diffusion coefficient of biopolymers within the mesh). Having higher water content in the cell, biomolecules diffuse faster as they are diluted, leading to an increase of diffusion coefficient and subsequent decrease of $\tau_{1}$ (Miermont et al., 2013; Moeendarbary et al., 2013). Secondly, the power-law relaxation α is relating to the structure changes of the cytoskeleton, which characterizes the fluidity of the cytoskeleton network within a range from 0 to 1 (Hecht et al., 2015). α=0 corresponds to a purely elastic solid; since it does not relax with time. α=1 corresponds to a purely viscous Newtonian fluid (Hecht et al., 2015). The increase of α indicates more liquid-like behavior of the cell skeleton, i.e., moisturization of the cell. Thus, the correlated changes of four parameters provide the state of moisturization in skin keratinocyte.

The parameters, *τ*_1_, *α*, *E*_0_, and *E*_1_, were measured in cultured keratinocytes after treatment of EBN extract and digest. These parameters were compared to values measured for keratinocytes under hypotonic and hypertonic conditions. For keratinocytes under hypotonic condition, simultaneous decreases of *τ*_1_, *E*_*0*_, and *E*_*1*_, as well as increase of α, were identified, which clearly showed the moisturization of cells. In contrast, the hypertonic condition caused opposite changes of these parameters implying dehydration of cells (Figure 11D). The correlated statistically significant changes of the four parameters in EBN-treated keratinocytes indicated that EBN extract and digest could promote the moisturization of cells, similar to cell behavior under hypotonic environment (Figure 11D). The 24-h treatment of EBN digest caused better changes of α and E_1_ values than that of EBN extract, which suggested that the cell skeleton became more fluid-like and more water was retained in the cytosol. Thus, the analysis by AFM further validated the moisturizing effect of EBN digest in cultured keratinocytes from the perspective of cell mechanics.

## Discussion

In Southeast Asian countries, EBN has been known for its ability to prevent lung diseases, strengthen the immune system, and improve complexion. Epidermal growth factor (EGF) has been found in EBN, which has proposed to correspond the proliferation effect of EBN in epidermal tissues (Kong et al., 1987). In addition, N-acetylneuraminic acid, contained in EBN, possessed a skin-whitening function (Chan et al., 2015), and additionally EBN was shown to reduce water loss, wrinkle area, and dermal thickness of skin (Terazawa and Shimoda, 2020). Although there are many claims on the skin-promoting effects and medicinal values of EBN, there has been very little scientific evidence showing the functions, and/or the active ingredients, of EBN in moisturizing skin. Here, we have provided different evidence to reveal the signaling pathway of EBN extract and digest in regulating the expressions of filaggrin and filaggrin-2, two important skin barrier proteins of the SFTP family playing roles in water balance of skin surface. The EBN-mediated regulation of filaggrin and filaggrin-2 is demonstrated to be triggered by p38-MAPK signaling pathway and various transcriptional factors, e.g. GATA3, PPARα, PPARβ, and PPARγ.

The epidermis composes of four layers: stratum basale, stratum spinosum, stratum granulosum, and stratum corneum from bottom to top. Keratinocytes are proliferative in the basal layer and then keratinized, or differentiated, from the spinous to cornified layers; whereas the Ca^2+^ concentration increases as it moves up the layers (Bikle, 2018). The increase in extracellular Ca^2+^ concentration facilitates the exocytosis of lipid-containing lamellar bodies and the proteolysis of filaggrin into NMFs, which function as retaining water and maintaining elasticity in stratum corneum. The enzymatic digestion of EBN was devised to enhance the protein extraction rate, as well as to increase the release of sialic acid from EBN (Wong et al., 2018a). In the enzymatic digestion, pepsin in simulated gastric fluid was used to mimic the gastric digestion environment in the human body and to maximize the digestion of the large proteins contained in EBN. As shown here, the digested product of EBN significantly increased filaggrin and filaggrin-2 expressions at mRNA and protein levels, dose-dependently. This suggests that the enzymatic digestion can release small functional peptides from EBN that specifically increase the gene expression. Additionally, EBN digest showed better efficiency, as compared to EBN extract, in increasing the protein expressions. Niacinamide, vitamin B3, is a well-known barrier repair vehicle widely used as a moisturizer and body wash ingredient (Del Rosso, 2011), as well as a known PPAR agonist in enhancing filaggrin biosynthesis (Harding and Rawlings, 2000). According to Nisbet et al. (2019), 0.1% niacinamide induced a nearly 3-fold change of filaggrin mRNA level in HaCaT keratinocytes. Thus, the moisturizing effect of digested EBN in protecting skin barrier is comparable to that of niacinamide.

In addition to transcriptional regulation, we suspect that EBN could function in regulating the post-translation modification of filaggrin and filaggrin-2. The proteolysis and deimination of profilaggrin and filaggrin-2 are promoted by proteases caspase-14 (CASP14) (Hoste et al., 2011), protein arginine deiminase 1 (PADI1) and 3 (PADI3) (Nachat et al., 2005), and calpain 1 (Sandilands et al., 2009; Hsu et al., 2011). At least as shown here, EBN triggered the expression of CSAP14, which could account for part of the moisturizing effect. The moisturizing factors are essential for stratum corneum hydration, barrier homeostasis, desquamation, and plasticity (Robinson et al., 2010), and therefore the epidermal hydration can be reserved. Loss-of-function mutation in filaggrin gene could reduce the level of NMFs (Kezic et al., 2008), and thereafter the skin could not maintain its hydration level and experience greater water loss. CASP14 is a cysteinyl aspartate specific proteinase confined in cornified epithelium playing a role in epidermal differentiation and NMF formation (Hoste et al., 2011; Masse et al., 2014). Two classes of widely used skin moisturizing ingredients, Cer-2 and Cer-6 ceramides, have been reported to stimulate CASP14 gene expression (Jiang et al., 2013). Having the EBN-induced CASP14 expression here, we suggest that EBN has the potential in developing into a moisturizer ingredient in cosmetics application.

Apart from the downstream pathway of filaggrin and filaggrin-2, the upstream signaling pathways regulating the moisturizing proteins are also important. The inhibition of p38-MAPK signaling pathway has been illustrated to abolish the expression of filaggrin in human epidermal keratinocytes (Nagosa et al., 2017). As demonstrated here with HaCaT keratinocytes, EBN significantly activated the phosphorylation of p38, and therefore the expressions of filaggrin and filaggrin-2 were mediated *via* the p38-MAPK signaling. The over expression of GATA3 in regulating the expressions of filaggrin and filaggrin-2 in human keratinocytes has been demonstrated (Zeitvogel et al., 2017). Here in keratinocytes, EBN significantly increased GATA3 expression, as well as the nuclear translocation of phosphorylated GATA3: these events promoted the expressions of filaggrin and filaggrin-2. Besides, the activation of PPAR expression and its nuclear translocation could also lead to an increased expression of filaggrin (Harding and Rawlings, 2000). The three PPAR isotypes: PPARα, PPARβ, and PPARγ, are all expressed in human skin, playing roles in skin homeostasis (Michalik and Wahli, 2007). EBN, both extract and digest, significantly increased the mRNA and protein levels of PPARs.

Biochemical assays, including western blot and real-time PCR, require an extraction process and disrupt cellular structures, which cannot provide information of EBN in physical appearance of keratinocyte. Thus, we have employed AFM, serving as a novel and effective technique, to reveal the moisturization state of keratinocyte under the treatment of EBN. In our AFM assays, the relaxation modulus E(t) obtained from the mechanical measurements describes how cells behave under mechanical perturbation, which is closely related to the moisturization state of living cells by $E_{0},E_{1},\tau_{1}$, and α. By comparing the mechanics between EBN-treated cells and mechanical-induced moisturized or dehydrated cells, we can identify the moisturizing effect of EBN mechanically. The biochemical assay and mechanical characterization are in parallel to confirm the moisturization effects of EBN.

The developed digestion on EBN greatly releases free sialic acids and small functional peptides that could specifically increase the skin moisturizing effect. In our preliminary study, N-acetylneuraminic acid, the most abundant sialic acid contained in EBN, significantly induced the expression of filaggrin in cultured keratinocytes. Besides, the digested peptides of EBN have been fractionated by molecular sizing on a HPLC, and the fractions were probed for testing in inducing filaggrin and filaggrin-2. The fraction containing small peptides of molecular weight <1.7 kDa showed robust induction in expressions of filaggrin and filaggrin-2 (Supplementary Figure 1). These lines of evidence support the superior efficacy of EBN digest in moisturizing skin. Generally speaking, the large polypeptides provide limited therapeutic values due to their low membrane permeability, rapid degradation, and native allergen reactivity (Lindgren et al., 2000; Aguilar-Toalá et al., 2019). Recent report has shown that the food-derived peptides could exert better biological functions, as compared to their native proteins (Aguilar-Toalá et al., 2019). In the application of cosmetics products, low molecular weight peptides possess the advantages of higher bioavailability, deeper skin penetration, lower toxicity, and hypo-allergenicity (Aguilar-Toalá et al., 2019). On the other hand, most of the active compounds in current intradermally-injected or topical moisturizer formulations are synthetic chemicals, phytochemicals, metabolites, minerals, or vitamins: these agents may cause side effects, such as skin allergic reactions including irritation or inflammation. In a case reported by Lupton and Alster, (2000), continuous intra-dermal injections of hyaluronic acid gel led to bruising, erythema, and acute red nodules in treatment areas. In contrast, EBN-derived short peptides possess the advantages of lower toxicity and hypo-allergenicity. Furthermore, our preliminary results show that the digested EBN could ameliorate TNF-α induced dermatitis in keratinocytes (data not shown), which supports a greater potential of EBN in developing into anti-inflammatory and moisturizing skincare products.

## Data Availability

The original contributions presented in the study are included in the article/Supplementary Material, further inquiries can be directed to the corresponding author.
