# Supplementary figures and images for: Edible Bird’s Nest, an Asian Health Food Supplement, Possesses Moisturizing Effect by Regulating Expression of Filaggrin in Skin Keratinocyte
Source: Front Pharmacol. 2021 Jul 20;12:685982. doi: 10.3389/fphar.2021.685982 (PMC8329658; doi:10.3389/fphar.2021.685982)

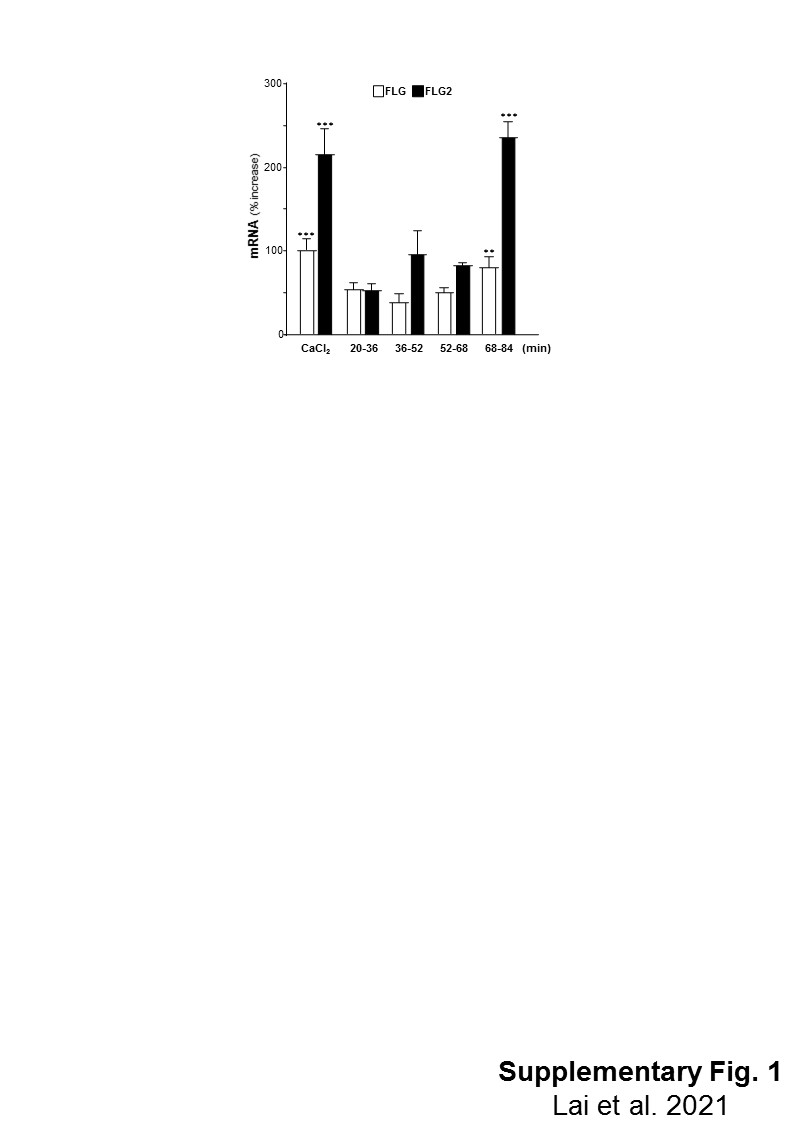

Supplement: Supplementary file 1 [file Image1.jpg]
